# Correction: Hernández-Pedro et al. Impact of Tyrosine Kinase Inhibitors on the Immune Response to SARS-CoV-2 Vaccination in Patients with Thoracic Malignancies. *Vaccines* 2023, *11*, 1612

**DOI:** 10.3390/vaccines13090913

**Published:** 2025-08-28

**Authors:** Norma Hernández-Pedro, Marisol Arroyo-Hernández, Pedro Barrios-Bernal, Eunice Romero-Nuñez, Victor A. Sosa-Hernandez, Santiago Ávila-Ríos, José Luis Maravillas-Montero, Rogelio Pérez-Padilla, Diego de Miguel-Perez, Christian Rolfo, Oscar Arrieta

**Affiliations:** 1 Laboratorio de Medicina Personalizada, Instituto Nacional de Cancerología, S.S.A., San Fernando 22 Sección XVI, Tlalpan, Mexico City 14080, Mexico; nhernandezp@incan.edu.mx (N.H.-P.); pedrobarrios@ciencias.unam.mx (P.B.-B.); eunice.romero@gmail.com (E.R.-N.); 2 Thoracic Oncology Unit, Instituto Nacional de Cancerología, S.S.A., San Fernando 22 Sección XVI, Tlalpan, Mexico City 14080, Mexico; marisol.neumologia@gmail.com; 3 Red de Apoyo a la Investigación, Instituto Nacional de Ciencias Médicas y Nutrición Salvador Zubirán, Mexico City 14080, Mexico; md.victor.sosa@gmail.com (V.A.S.-H.); maravillas@cic.unam.mx (J.L.M.-M.); 4 Centro de Investigación en Enfermedades Infecciosas (CIENI), Instituto Nacional de Enfermedades Respiratorias, Calzada de Tlalpan 4502, Belisario Domínguez Sección XVI, Tlalpan, Mexico City 14080, Mexico; santiago.avila@cieni.org.mx; 5 Department of Research on Tobacco and COPD, Instituto Nacional de Enfermedades Respiratorias, Calzada de Tlalpan 4502, Belisario Domínguez Sección XVI, Tlalpan, Mexico City 14080, Mexico; perezpad@unam.mx; 6 Mount Sinai Health System, Icahn School of Medicine at Mount Sinai, New York, NY 11776, USA; diego.demiguelperez@mssm.edu (D.d.M.-P.); christian.rolfo@mssm.edu (C.R.)

The authors would like to make the following corrections to this published paper [1].

The title of the paper should be updated from “Impact of Tyrosine Kinase Inhibitors on the Immune Response to SARS-CoV-2 Vaccination in Patients with Non-Small Cell Lung Cancer” to “Impact of Tyrosine Kinase Inhibitors on the Immune Response to SARS-CoV-2 Vaccination in Patients with Thoracic Malignancies”, to describe all included patients.

The authors would like to clarify that all individuals receiving the CanSino vaccine at the time of this study received only one dose. Therefore, to avoid confusion, in the second paragraph of Section 2.1. “Study Design and Participants”, the sentence “…or inactivated virus vaccines like Sinovac-CoronaVac in one or two doses according to the pre-established scheme” is changed to “…or inactivated virus vaccines like Sinovac-CoronaVac in one (CanSino) or two doses (all others) according to the pre-established scheme.” In the fifth paragraph of Section 2.1. “Study Design and Participants”, the second sentence “Blood samples were collected 30 days after the second dose of the COVID-19 vaccine to…” is changed to “Blood samples were collected 30 days after scheme completion of the COVID-19 vaccine to…”.

As currently published, some proportions were not accurately located within each section in Table 2. Moreover, additional changes were made to subtitles to improve their clarity. The title of Table 2 has been updated to “Clinical features of thoracic malignancy patients stratified by SARS CoV-2 antigen load and titles” to describe all included patients. The correct Table 2 appears below.

**Table 2:** Clinical features of thoracic malignancy patients stratified by SARS CoV-2 antigen load and titles.

Clinical Characteristics	SARS-COV-2 Antigen (>20)	SARS-COV-2 Antigen (<20)	*p*-Value	SARS-COV-2 Titles (−)	SARS-COV-2 Titles (+)	*p*-Value
N = 92	% (n)	% (n)		% (n)	% (n)	
**Sex**						
Male: 39	46.2 (18)	53.8 (21)		46.2 (18)	53.8 (21)	
Female: 53	45.3 (24)	54.7 (29)	0.934	50.9 (27)	49.1 (26)	0.650
**Age**						
<59: 41	34.1 (14)	65.9 (27)		39.0 (16)	61.0 (25)	
≥59: 51	54.9 (28)	45.1 (23)	0.047	56.9 (29)	43.1 (22)	0.089
**Smoking status**						
No: 67	41.8 (28)	58.2 (39)		46.3 (31)	53.7 (36)	
Yes: 25	56.0 (14)	44.0 (11)	0.224	56.0 (14)	44.0 (11)	0.406
**Woodsmoke exposure**						
No: 72	48.6 (35)	51.4 (37)		50.0 (36)	50.0 (36)	
Yes: 20	35.0 (7)	65.0 (13)	0.280	45.0 (9)	55.0 (11)	0.692
**Asbestos exposure**						
No: 82	47.6 (39)	52.4 (43)		50.0 (41)	50.0 (41)	
Yes: 10	30.0 (3)	70.0 (7)	0.336 *	40.0 (4)	60.0 (6)	0.740 *
**ECOG PS**						
0–1: 86	47.7 (41)	52.3 (45)		51.2 (44)	48.8 (42)	
>2: 6	16.7 (1)	83.3 (5)	0.214 *	16.7 (1)	83.3 (5)	0.204 *
**Clinical stage**						
I–III: 10	60.0 (6)	40.0 (4)		60.0 (6)	40.0 (4)	
IV: 82	43.9 (36)	56.1 (46)	0.503 *	47.6 (39)	52.4 (43)	0.518 *
**Histology**						
Adenocarcinoma: 79	44.3 (35)	55.7 (44)		48.1 (38)	51.9 (41)	
Squamous: 4	75.0 (3)	25.0 (1)		75.0 (3)	25.0 (1)	
Mesothelioma: 5	20.0 (1)	80.0 (4)		20.0 (1)	80.0 (4)	
Others: 4	75.0 (3)	25.0 (1)	0.245	75.0 (3)	25.0 (1)	0.276
**Histological grade**						
Low: 15	46.7 (7)	53.3 (8)		53.3 (8)	46.7 (7)	
Intermediate: 27	51.9 (14)	48.1 (13)		51.9 (14)	48.1 (13)	
High: 23	34.8 (8)	65.2 (15)		34.8 (8)	65.2 (15)	
n/a: 14	42.9 (6)	57.1 (8)	0.679	57.1 (8)	42.9 (6)	0.492
**Metastases**						
CNS: 23	47.8 (11)	52.2 (12)	0.809	52.2 (12)	47.8 (11)	0.718
Liver: 7	42.9 (3)	57.1 (4)	1.00 *	42.9 (3)	57.1 (4)	1.00 *
Lung: 60	48.3 (29)	51.7 (31)	0.480	51.7 (31)	48.3 (29)	0.469
Ganglia: 8	62.5 (5)	37.5 (3)	0.317 *	62.5 (5)	37.5 (3)	0.481 *
Bone: 28	35.7 (10)	64.3 (18)	0.206	42.9 (12)	57.1 (16)	0.442
Pulmonary effusion: 31	51.6 (16)	48.4 (15)	0.413	58.1 (18)	41.9 (13)	0.211
**EGFR**						
Wild type: 54	44.4 (24)	55.6 (30)		46.3 (25)	53.7 (29)	
Mutant: 38	47.4 (18)	52.6 (20)	0.782	52.6 (20)	47.4 (18)	0.549
**EGFR subtype**						
Exon 19 del: 26	42.3 (11)	57.7 (15)	0.686	46.2 (12)	53.8 (14)	0.740
Exon 21 L858R: 12	50.0 (6)	50.0 (6)	0.746	58.3 (7)	41.7 (5)	0.547 *
Exon 20 T790M: 1	100 (1)	0.0 (0)	0.457 *	100 (1)	0.0 (0)	0.489 *
**ALK**						
Wild type: 68	51.5 (35)	48.5 (33)		54.4 (37)	45.6 (31)	
Mutant: 24	29.2 (7)	70.8 (17)	0.059	33.3 (8)	66.7 (16)	0.076
**TKI treatment**						
No: 20	55.0 (11)	45.0 (9)		55.0 (11)	45.0 (9)	
Yes: 55	40.0 (22)	60.0 (33)	0.247	45.5 (25)	54.5 (30)	0.464
**QT treatment**						
No: 61	41.0 (25)	59.0 (36)		45.9 (28)	54.1 (33)	
Yes: 31	54.8 (17)	45.2 (14)	0.207	54.8 (17)	45.2 (14)	0.418
**Vaccine**						
BNT162b2: 31	48.4 (15)	51.6 (16)		48.4 (15)	51.6 (16)	
AZD1222: 37	45.9 (17)	54.1 (20)		48.6 (18)	51.4 (19)	
Sputnik: 10	50.0 (5)	50.0 (5)		50.0 (5)	50.0 (5)	
Sinovac: 13	38.5 (5)	61.5 (8)		46.2 (6)	53.8 (7)	
Cansino: 1	0.0 (0)	100 (1)	0.864	100 (1)	0 (0)	0.895
**COVID-19**						
No: 82	47.6 (39)	52.4 (43)		51.2 (42)	48.8 (40)	
Yes: 10	30.0 (3)	70.0 (7)	0.336 *	30.0 (3)	70.0 (7)	0.317 *

* Fisher’s exact test. All other comparisons were performed using the chi-square test.

In Section 3.1 “Clinical Characteristics”, some proportions did not match between the text and Tables 1 and 2. Corrections have been made here. Specifically, the fourth and fifth sentences were updated to “In the lung cancer cohort, 89.2% (n = 82) of patients had IV-stage disease, and only 10.8% (n = 10) were early-stage. Adenocarcinoma was the most common type of cancer in the overall sample, 85.9% (n = 79), while mesothelioma, 5.4% (n = 5), squamous cell carcinoma, 4.3% (n = 4), and other, 4.3% (n = 4), were found in a smaller proportion of cases (Table 2)”. The citation to Table 2 at the end of this paragraph was removed and added in the fifth sentence.

The paragraph in Section 3.2 “SARS-CoV-2 Vaccines Distribution” should be changed to the following after a detailed review of data:

“In the healthy cohort, 33.3% (n = 9) of subjects received Oxford/AstraZeneca (AZD1222), 33.3% (n = 9) Pfizer-BioNTech (BNT162b2), 18.5% (n = 5) Johnson & Johnson’s Janssen, 11.1% (n = 3) Sputnik V, and 3.7% (n = 1) Sinovac. In the meantime, NSCLC patients received AZD1222 in 40.2% (n = 37), BNT162b2 in 33.7% (n = 31), Sinovac in 14.1% (n = 13), Sputnik V in 10.9% (n = 10), and CanSino in only 1.1% (n = 1) of cases (Table 1).”

The title of Table 3, “SARS-CoV-2 vaccine-related adverse effects in NSCLC patients according to Common Terminology Criteria for Adverse Events (CTCAE v5.0) scale”, must be modified to the following version according to the Materials and Methods: “SARS-CoV-2 vaccine-related adverse effects in NSCLC patients according to the Mexican Official Standard for epidemiological surveillance [12]”, as this was the scale used for measuring vaccine-related adverse effects, and to maintain methodological precision and consistency. Additionally, ‘SARS-CoV-2 Neutralizing Antibodies’ was added as a column header to accurately describe the detection methodology underlying these measurements. The newly added ref. [12] and correct Table 3 appear below.

12. Tapia-Conyer, R.; Kuri-Morales, P.; González-Urbán, L.; Sarti, E. Evaluation and Reform of Mexican National Epidemiological Surveillance System. *Am. J. Public Health* **2001**, *91*, 1758–1760. https://doi.org/10.2105/ajph.91.11.1758.

**Table 3:** SARS-CoV-2 vaccine-related adverse effects in NSCLC patients according to the Mexican Official Standard for epidemiological surveillance [12].

Clinical Characteristics	Neutralization Antibodies for SARS-COV-2 Virus (Cut-Off > 20)	Neutralization Antibodies for SARS-COV-2 Virus (Cut-Off < 20)	*p*-Value	SARS-COV-2 Antibodies Titles (−)	SARS-COV-2 Antibodies Titles (+)	*p*-Value
N = 92	% (n)	% (n)		% (n)	% (n)	
**Sex**						
Male: 39	46.2 (18)	53.8 (21)		46.2 (18)	53.8 (21)	
Female: 53	45.3 (24)	54.7 (29)	0.934	50.9 (27)	49.1 (26)	0.678
**Age**						
<59: 41	34.1 (14)	65.9 (27)		39.0 (16)	61.0 (25)	
≥59: 51	54.9 (28)	45.1 (23)	0.047	56.9 (29)	43.1 (22)	0.098
**Smoking status**						
No: 67	41.8 (28)	58.2 (39)		46.3 (31)	53.7 (36)	
Yes: 25	56.0 (14)	44.0 (11)	0.224	56 (14)	44.0 (11)	0.485
**Woodsmoke exposure**						
No: 72	48.6 (35)	51.4 (37)		50.0 (36)	50.0 (36)	
Yes: 20	35.0 (7)	65.0 (13)	0.280	45.0 (9)	55.0 (11)	0.802
**Asbestos exposure**						
No: 82	47.6 (39)	52.4 (43)		50 (41)	50 (41)	
Yes: 10	30.0 (3)	70.0 (7)	0.293	40 (4)	60 (6)	0.740
**ECOG PS**						
0–1: 86	47.7 (41)	52.3 (45)		51.2 (44)	48.8 (42)	
>2: 6	16.7 (1)	83.3 (5)	0.214	16.7 (1)	83.3 (5)	0.204
**Clinical stage**						
I–III: 10	60.0 (6)	40.0 (4)		60.0 (6)	40.0 (4)	
IV: 82	43.9 (36)	56.1 (46)	0.503	47.6 (39)	52.4 (43)	0.518
**Histology**						
Adenocarcinoma: 79	44.3 (35)	55.7 (44)		48.1 (38)	51.9 (41)	
Squamous: 4	75.0 (3)	25.0 (1)		75.0 (3)	25.0 (1)	
Mesothelioma: 5	20.0 (1)	80.0 (4)		20.0 (1)	80.0 (4)	
Others: 4	75.0 (3)	25.0 (1)	0.245	75.0 (3)	25.0 (1)	0.276
**Histological grade**						
Low: 15	46.7 (7)	53.3 (8)		53.3 (8)	46.7 (7)	
Intermediate: 27	51.9 (14)	48.1 (13)		51.9 (14)	48.1 (13)	
High: 23	34.8 (8)	65.2 (15)		34.8 (8)	65.2 (15)	
n/a: 14	42.9 (6)	57.1 (8)	0.679	57.1 (8)	42.9 (6)	0.492
Metastases *						
CNS: 23	47.8 (11)	52.2 (12)	0.809	52.2 (12)	47.8 (11)	0.811
Liver: 7	42.9 (3)	57.1 (4)	0.877	42.9 (3)	57.1 (4)	1.00
Lung: 60	48.3 (29)	51.7 (31)	0.480	51.7 (31)	48.3 (29)	0.517
Ganglia: 8	62.5 (5)	37.5 (3)	0.317	62.5 (5)	37.5 (3)	0.481
Bone: 28	35.7 (10)	64.3 (18)	0.206	42.9 (12)	57.1 (16)	0.501
Pulmonary effusion: 31	51.6 (16)	48.4 (15)	0.413	58.1 (18)	41.9 (13)	0.271
**EGFR**						
Wild type: 54	44.4 (24)	55.6 (30)		46.3 (25)	53.7 (29)	
Mutant: 38	47.4 (18)	52.6 (20)	0.782	52.6 (20)	47.4 (18)	0.672
**EGFR subtype**						
Exon 19 del: 26	42.3 (11)	57.7 (15)	0.686	46.2 (12)	53.8 (14)	0.819
Exon 21 L858R: 12	50.0 (6)	50.0 (6)	0.746	58.3 (7)	41.7 (5)	0.547
Exon 20 T790M: 1	100 (1)	0.0 (0)	0.273	100 (1)	0.0 (0)	0.489
**ALK**						
Wild type: 68	51.5 (35)	48.5 (33)		54.4 (37)	45.6 (31)	
Mutant: 24	29.2 (7)	70.8 (17)	0.059	33.3 (8)	66.7 (16)	0.098
**TKI treatment**						
No: 20	55.0 (11)	45.0 (9)		55.0 (11)	45.0 (9)	
Yes: 55	40.0 (22)	60.0 (33)	0.247	45.5 (25)	54.5 (30)	0.602
**QT treatment**						
No: 61	41.0 (25)	59.0 (36)		45.9 (28)	54.1 (33)	
Yes: 31	54.8 (17)	45.2 (14)	0.207	54.8 (17)	45.2 (14)	0.509
**Vaccine**						
BNT162b2: 31	48.4 (15)	51.6 (16)		48.4 (15)	51.6 (16)	
AZD1222: 36	45.9 (17)	54.1 (20)		48.6 (18)	51.4 (19)	
Sputnik: 10	50.0 (5)	50.0 (5)		50.0 (5)	50.0 (5)	
Sinovac: 13	38.5 (5)	61.5 (8)		46.2 (6)	53.8 (7)	
Cansino: 1	0.0 (0)	100 (1)	0.864	100 (1)	0 (0)	0.895
**COVID-19**						
No: 82	47.6 (39)	52.4 (43)		51.2 (42)	48.8 (40)	
Yes: 10	30.0 (3)	70.0 (7)	0.293	30.0 (3)	70.0 (7)	0.317

* Each patient can have more than one metastatic site, resulting in a total of 157 in all 92 patients.

To enhance the clarity of the proportions described in all tables, a flow diagram was created to display the selection process of patients for ELISA and flow cytometry assays (Supplementary Figure S4).

**Figure S4:**
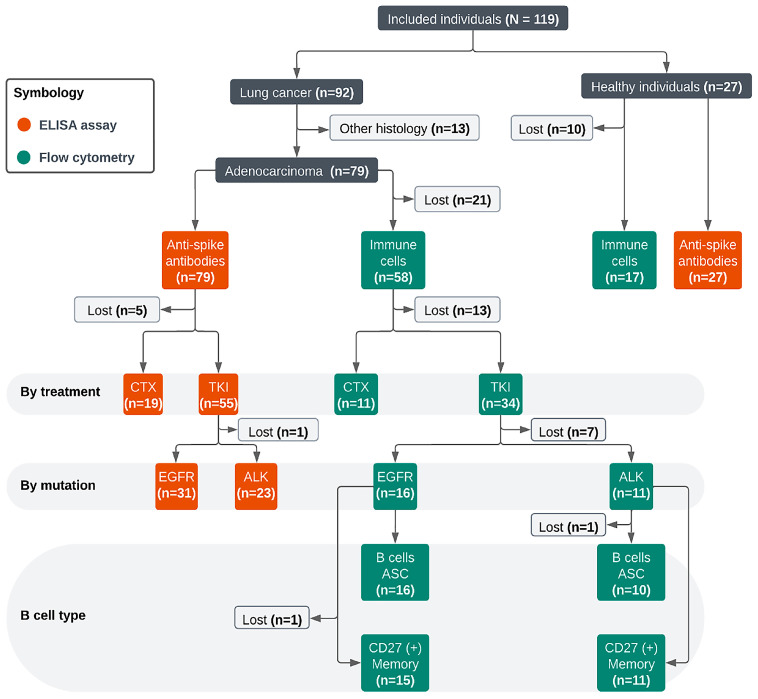
Flow diagram of individuals included for analysis: CTX, chemotherapy. TKI, tyrosine kinase inhibitor. EGFR, epidermal growth factor receptor. ALK, anaplastic lymphoma kinase. CD27, cluster of differentiation 27. ASC, antibody-secreting cell. B cells, B lymphocytes.

The authors state that the scientific conclusions are unaffected. This correction was approved by the Academic Editor. The original publication has also been updated.
