# Supplementary material for: Impact of Tyrosine Kinase Inhibitors on the Immune Response to SARS-CoV-2 Vaccination in Patients with Non-Small Cell Lung Cancer
Source: Vaccines (Basel). 2023 Oct 19;11(10):1612. doi: 10.3390/vaccines11101612 (PMC10611320; doi:10.3390/vaccines11101612)
Supplement: Supplementary file 1 [file vaccines-11-01612-s001.zip › vaccines-2587192-supplementary.pdf]

**Supplementary material.**

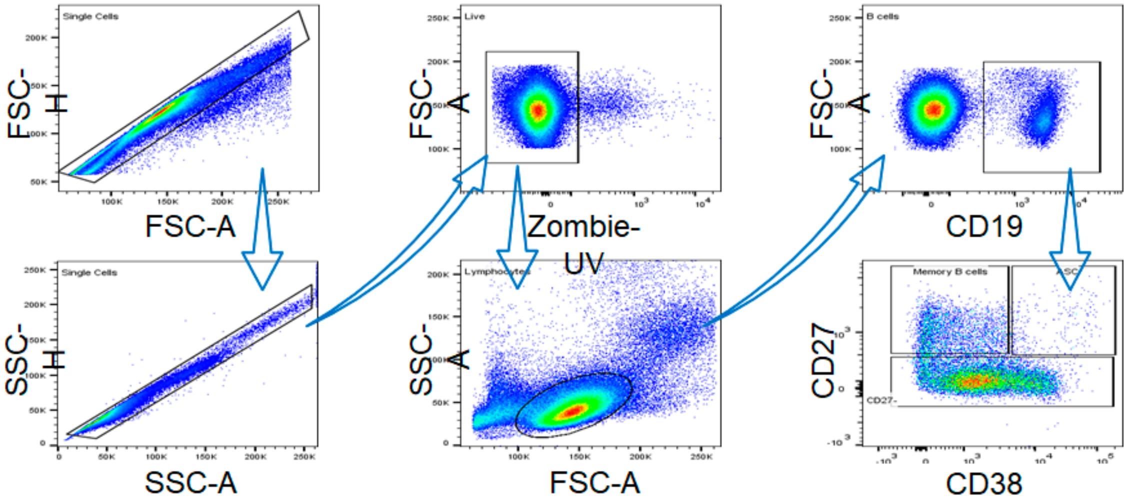


| Antibody/reagent | Emission peak | Marker Type |
|---|---|---|
| APC anti-human CD19 | 660 nm | Pan B cell |
| PE anti-human CD27 | 574 nm | Memory B, Plasmablast, Plasma cell |
| FITC anti-human CD38 | 525 nm | Immature B, Germinal Center, Plasmablast, Plasma cell |
| Zombie-UV | 465 nm | Fixable Viability Dye |

**Figure S1.** Flow Cytometry-Based Population Analysis and Corresponding Population Marker Emission Peaks.

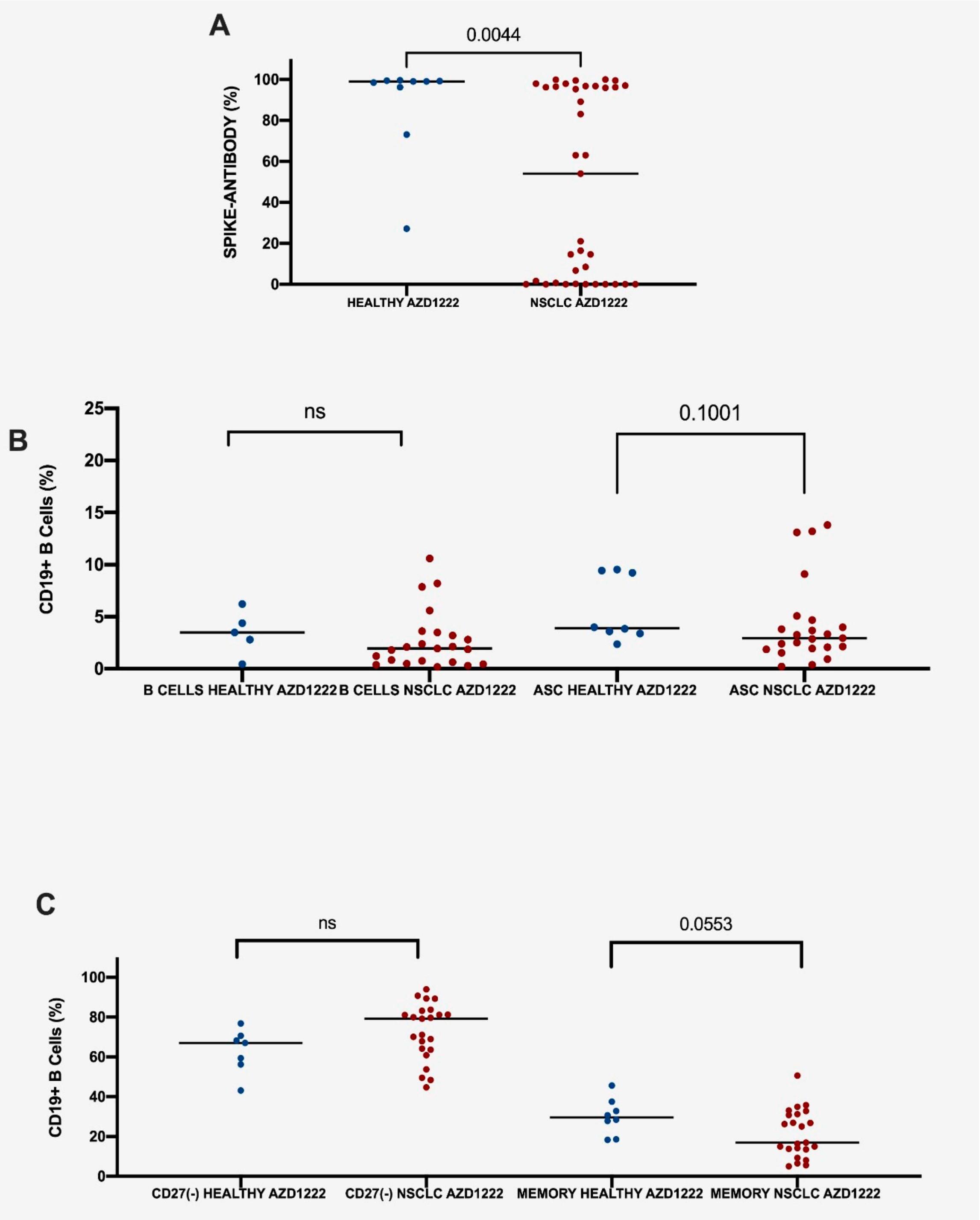


**Figure S2.** Percentage of spike-antibodies between healthy and NSCLC groups that received AZD1222 vaccine. (**A**) Percentage of spike-antibodies between all healthy and NSCLC patients. (**B**) Percentage of CD19+ B cells and ASC between healthy and NSCLC groups. (**C**) CD27(-) and memory CD19+ B cells between healthy and NSCLC groups.

Statistical significance: *p* value ≤0.05. NSCLC, Non-Small Cell Lung Cancer. ASC, Antibody-Secreting Cells. Ns, non-significant. AZD1222, Oxford-Astra Zeneca vaccine.

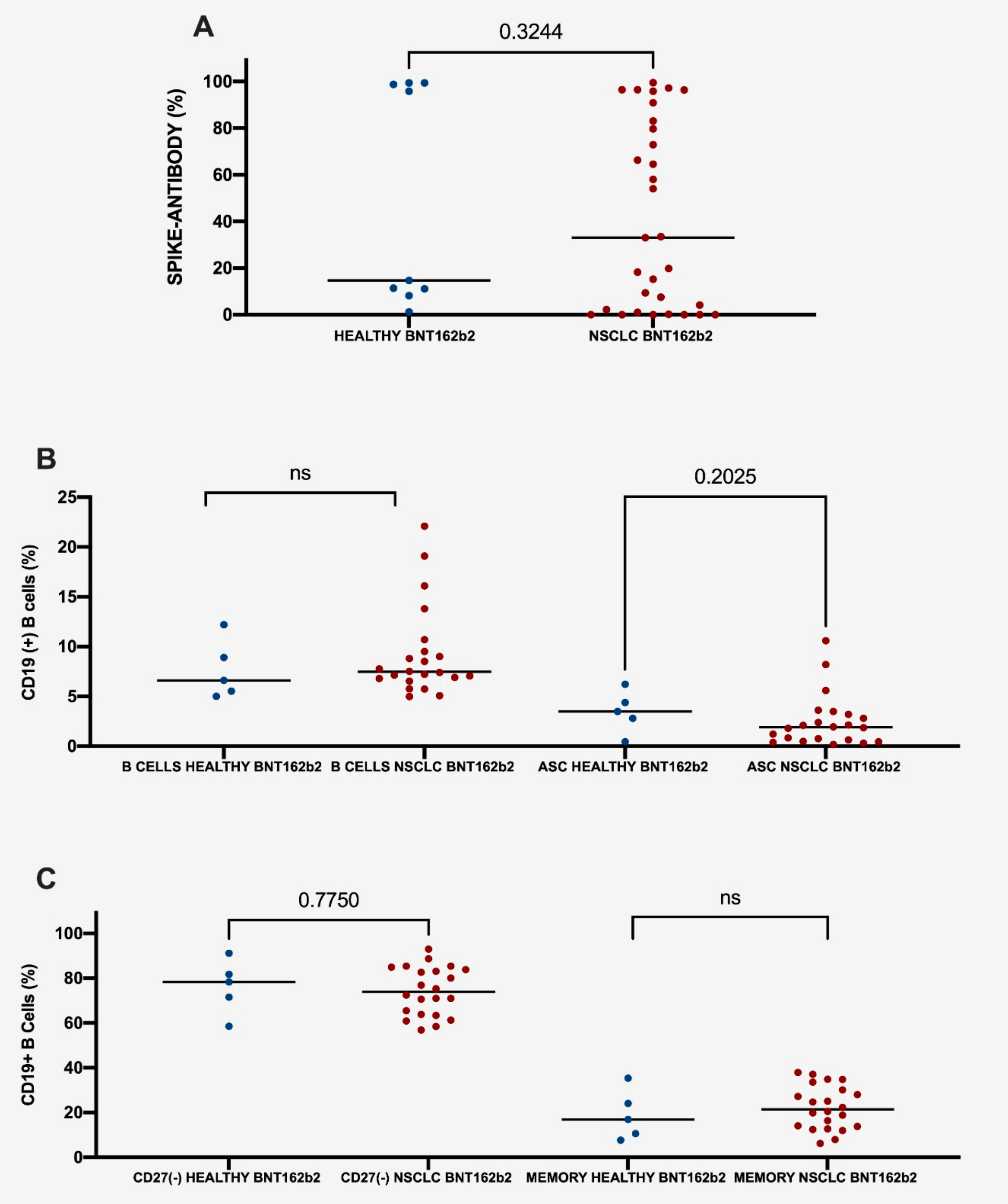


**Figure S3.** Percentage of spike-antibodies between healthy and NSCLC groups that received BNT162b2 vaccine. (**A**) Percentage of spike-antibodies between all healthy and NSCLC patients. (**B**) Percentage of CD19+ B cells and ASC between healthy and NSCLC groups. (**C**) CD27(-) and memory CD19+ B cells between healthy and NSCLC groups. Statistical significance: *p* value ≤0.05. NSCLC, Non-Small Cell Lung Cancer. ASC, Antibody-Secreting Cells. BNT162b2, BioNTech-Pfizer vaccine.
